# A workflow to discover partial differential equations from data: Application to the dynamics of tree biomass

**DOI:** 10.1016/j.mex.2025.103560

**Published:** 2025-08-17

**Authors:** Emilie Peynaud, Paulin Melatagia, Serge Stinckwich, Jean-François Barczi

**Affiliations:** a CIRAD, UMR AMAP, F-34398 Montpellier, France; b AMAP, Univ Montpellier, CIRAD, CNRS, INRAE, IRD, Montpellier, France; c Université de Yaoundé I, Cameroon, IRD, UMMISCO, F-93143, Bondy, France; d United Nations University Institute in Macau, Macau SAR, China

**Keywords:** Physics informed neural network, Parameter estimation, Data-driven modelling of plants, Theory-guided data model

## Abstract

Mixed data and theory driven methods are promising approaches that can be used to bring better understanding of complex dynamics in life sciences. For vegetation growth, integrated knowledge may be lacking to design theoretical models like partial differential equations (PDE). This lack can be complemented by using data. The method presented in this paper is a generic computational workflow called CEDI that aims at discovering PDE models from data. As an illustration, we tested the workflow on biomass dynamics of three different 3D trees of specific architectural types.

● The name CEDI represents the four steps composing the workflow: data Collection, Extrapolation, Differentiation and Identification.

● The originality of this workflow is twofold: first, it encompasses the whole modeling process from the definition of the variables to the design of a PDE, and second it has been designed to be generic in a sense that it can apply to any dynamics and it covers most existing data driven PDE discovering methods.

● The workflow offers a framework to better understand data driven PDE discovering methods and a tool for modeling any dynamics, provided that right data and knowledge and also good algorithm settings are available.

## Graphical abstract

**Figure:**
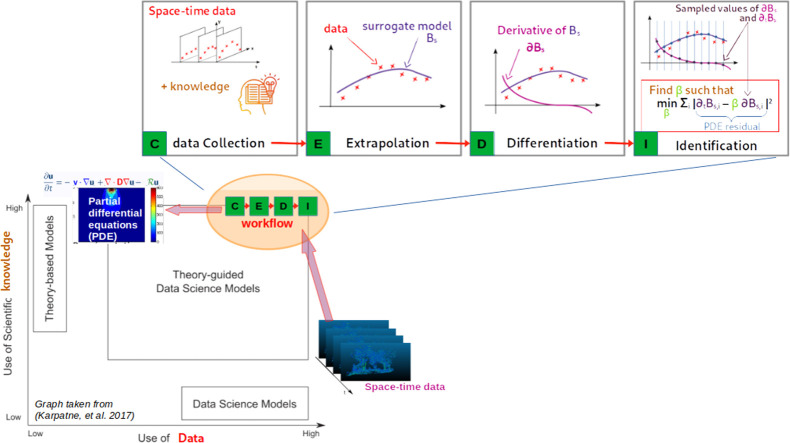

## Specifications table

**Table:** 

**Subject area**	Bioinformatics
**More specific subject area**	Partial differential equations and neural networks
**Name of your method**	Workflow for data Collection, Extrapolation, Differentiation and Identification for partial differential equation discovery
**Name and reference of original method**	Physic Informed neural network
**Resource availability**	Dataset: J.-F. Barczi [1], “Plant growth simulation data”, https://doi.org/10.18167/DVN1/VK0J16, CIRAD Dataverse, V1. Source code: https://gitlab.cirad.fr/amap/personal/emiliepeynaud/pydata2pde.git SoftWare Hash IDentifier: swh:1:dir:fd7172e67af912dddd7f2f50ccb6f42f7adb913f

## Background

Modeling approaches are crucial tools to characterize tree growth and dynamics in complex plant systems, such as cocoa-based agroforestry systems. Partial differential equations (PDE) are relevant models to represent space and time variations that govern the dynamics of any phenomenon, including those in life sciences. PDEs benefit from a well-established theory that mathematically legitimates this formalism. It applies to various space and time scales, which is a real advantage when dealing with plant and vegetation dynamics. However, estimation of parameters of PDE models still raises questions. The purpose of the workflow presented here is to characterize any dynamics by discovering the PDE that governs the underlying phenomenon using data. The conventional way to derive a PDE model is to formulate with mathematical expressions the known principles that govern the dynamics, considering a given set of assumptions. The workflow detailed in this paper gives an alternative within the paradigm of theory-guided data science [13] that uses data to compensate for a potential lack of knowledge on the principles governing trees’ dynamics.

Methods for discovering PDE models from data are gaining a growing interest especially in computational physics [7]. From measurements of the PDE variable at some points in space and time, these methods consist in computing additional estimates of the variable and its partial derivatives to build a library of candidate terms. Among this library, the methods then select a parsimonious combination of terms that best describes the dynamics of the variable. This identification step can be seen as a particular optimization problem which is solved thanks to techniques like statistical inferences [10], sparse regressions [17], sparse least square methods [18] or physic informed neural networks [3,16,19,6]. Most of the work cited here is focused on the optimization problem and is based on synthetic data computed from direct solving of a known PDE and it deals with application in physics where the constitutive laws and hence the PDE forms are well established [14]. This literature review showed that all cases shared common methods for addressing this PDE identification problem that may be encapsulated into a generic workflow providing guidelines for similar cases.

The way to discover PDE proposed in this paper takes the form of a workflow called CEDI and is designed to be generic and adjustable for applications where knowledge or data is lacking. Its originality lies in the embedding of the whole modeling process which is splitted into four formal steps: 1) the definition of the nature of the variable and the data collection (C), 2) the estimates of the values of the variable (E like extrapolation) and 3) its derivatives (D) and 4) the identification (I) of the PDE terms. This gives to the workflow its modularity and its genericity which are the main changes over existing works. For the sake of illustration and as a proof of concept, we applied the workflow to the discovery of PDE governing the biomass dynamics of growing trees from realistic data, using neural networks in the extrapolation and identification steps. The biomass dynamics are considered here as the variations of the leaf and wood mass over space and time. The application to trees as individuals or as communities is not straightforward, but our workflow can complement existing experimental and modeling approaches to better understand the dynamics of plants. The workflow may open new perspectives to characterize dynamics of plants through data by selecting the appropriate differential operators according to both knowledge and measurement of the dynamics. It may also help to give biological meaning to differential operators versus plants by performing successive runs on contrasted dataset.

## Method details

This section consists of two parts. The first one is an overview that describes the generic CEDI workflow, which is the main topic of this paper. The second one consists of a case study to apply the CEDI workflow to the specific domain of tree growth PDE modeling, providing a practical implementation to demonstrate and clarify the proposed workflow.

### Overview of the CEDI workflow and genericity

**Fig. 1:**
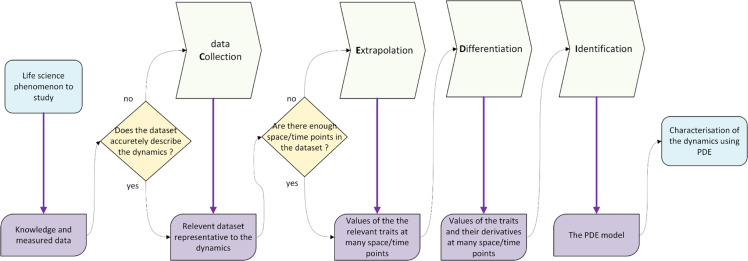
The CEDI workflow for PDE discovery from data.

The purpose of the generic workflow presented in this paper is to characterize any dynamics by discovering the PDE that governs the underlying phenomenon using data. By dynamics, we mean the way in which the system under study evolves over time and space under the influence of the processes to which it is subjected. Let us assume that the phenomenon of interest can be monitored or described through the measurement of a variable that varies with respect to time and space. We assume that this variable has sufficient regularity (such as continuity and differentiability) and satisfies a PDE defined in a bounded domain, together with appropriate initial and boundary conditions. The PDE also involves parameters that quantify the causality of the dynamics. The workflow consists in four main sequential actions performed on data that migrates from one step to the next: the data collection (C), the extrapolation (E), the computing of the partial derivatives (D) and the identification (I) of the PDE terms as illustrated in Fig. 1. The purposes of each of the four formal steps are defined below:

#### Data collection

The first step is to gather the knowledge available about the phenomenon according to the scientific question motivating the modeling process. Then one determines the traits that vary over space and time and that are meaningful with respect to the scientific question. Let us consider only one trait to simplify the presentation. This trait is mathematically represented by the variable of the PDE and its values must be amenable at various space and time points to build a dataset that faithfully shows the dynamics, as mentioned in Fig. 1. The construction of the dataset depends on the scientific fields and on the techniques available. For example, it may be built thanks to direct measurements of the trait or thanks to the reconstruction of chrono-sequences from a single observation. In this paper, we apply the workflow on measurements of biomass on simulated growing trees across time and space. The dataset may also be built thanks to automatic tools that extract a meaningful dataset from a large and heterogeneous database. Temporal and spatial sampling are supposed to guarantee the representativeness of the dynamics in a dataset of reasonable size and for a reasonable cost. How to know if the dataset faithfully describes the dynamics is a key question that has no general answer. For each case, it may be answered based on requests of both experts on the studied subject and experts on numerical processing.

#### Extrapolation

This step consists in the design of a surrogate model able to mimic the dynamics of the variable of interest to smooth or complete the data previously collected. Thanks to the surrogate model, one can compute estimates of the variable at any space-time point for example in cases of geographically missing data (if the sampling was not exhaustive enough across the experimental domain) or time missing data (if the sampling time step was not fine enough). This step requires the implementation of an interpolation method (polynomial, spline, neural networks) on which the next step is based for the computation of missing values of the variable but also estimates of the partial derivatives.

#### Differentiation or computation of the derivatives

Thanks to the surrogate model, the third step consists in computing estimated values of the partial derivatives at some space-time points, not necessarily those of the collected data, in order to build the library of candidate terms for the PDE (also called catalogue in the following). The candidate terms are chosen according to the available knowledge about dynamics. This selection may be based on domain knowledge or on empirical observations. The type of surrogate model often determines the method used to compute the derivatives.

#### Identification

The goal of the last step is to select the candidate terms of the PDE that best represent the dynamics and their degree of contribution to it. This is formulated as an optimization problem that minimizes a cost function defined as the residual of the PDE. Knowledge about dynamics helps to design the a priori form of the PDE made of a weighted sum of partial derivatives. If the set of partial derivatives involved in the PDE is known in advance, the identification step reduces to a parameter estimation problem where the goal is to estimate the weight of each partial derivative. If there is a lack of knowledge about dynamics, one can consider several partial derivatives as candidates (they will form a library of partial derivatives or catalogue). Then, selection methods allow us to select the limited number of terms of the PDE. The identification step requires special attention since it raises mathematical questions (well-posedness) as well as implementation and numerical questions (convergence), but also modeling questions (representativeness of the resulting PDE).

The main strength of the CEDI workflow lies in its generalizability in the sense that it encompasses every study about data-driven PDE extraction methods that we found in literature. This is illustrated in Table 1, where we have split some examples of these methods to fit the four steps of the CEDI workflow. These examples let us suppose that CEDI workflow will help any team to prevent and overpass standard issues that rise while discovering PDE from a dataset.

**Table 1:** Some examples of data-driven PDE extraction methods decomposed into the CEDI workflow.

	Data Collection (C)	Extrapolation (E)	Differentiation (D)	Identification (I)
[11]	Data computed on a grid by adding a Gaussian noise to the analytic solution of an advection equation. Real data application to the prices of SP-500 European call options	B-splines tensor product approximation introduced as a penalization term in the identification step	Differentiation based on the B-spline decomposition through the B-spline derivatives	Frequentist inference based on the maximisation of a penalized least square criterion and Bayesian inference where the frequentist least square criterion is translated into a prior distribution
[18]	Data sampled and noised on a grid from simulation performed by direct solving of the PDE on a higher resolution grid	Fourier transform	Spectral method based on the Fourier transform for the space derivatives, and first-order backward difference method for time derivatives	L1-regularized (sparse) least-square optimization problem solved with the Douglas-Rachford algorithm
[17]	Random sampling and noising of spatial points and uniform sampling for time points from simulated data by direct solving of the PDE	Polynomial interpolation	Finite difference technique applied to a localized points near each measurement points and based on the polynomial interpolation	Sequential threshold ridge regression (STRidge) consisting in a ridge regression problem with hard threshold
[16]	Random sub-sampling and noising of high resolution dataset generated by direct solving of the PDE	Deep neural network	Automatic differentiation (TensorFlow)	Training of the deep neural network (of the extrapolation step) with the L-BFGS algorithm and residual of the a priori known PDE included in the loss function
[3]	Measurement of temperature distribution from weather stations in Sweden. Data augmentation by linear interpolation in space and time. Scaling of the data.	Deep neural network trained with BFGS or L-BFGS algorithms	Back-propagation or automatic differentiation (TensorFlow or PyTorch)	Feedforward neural network trained with gradient based methods with L1 regularization for sparsity and feature selection techniques
[19]	Generated by direct solving of the PDE with Chebfun package in MATLAB. 1D in space with noise and limited data	Rational Neural Network (RatNN) with Adam and LBFGS optimizers	Autodiff capabilities provided by PyTorch	1) Rational Neural Network with Adam and LBFGS optimizers and re-sampling of the collocation points. 2) Sparse regression technique based on recursive feature elimination (RFE) algorithm

### Detailed presentation with application to tree biomass dynamics

In the following we detail the application of the workflow to the modeling of tree biomass dynamics for the sake of illustration of PDE discovering process from data. For this purpose we defined a very simple Multi Layer Perceptron (MLP) architecture strong enough to provide promising results. Further studies on the particular plant growth question may need more accurate neural networks for step E and I of CEDI and an improved dataset.

#### Data collection

Usually, the modeling process starts with the definition of a scientific question and state of the art on the subject. Here the goal is to illustrate the CEDI workflow, so we intentionally simplify what a tree is and how it behaves through space and time. Let us assume the following. The dynamics of tree growth can be seen as the result of continuous biomass creation through photosynthesis (occurring in the leaves) and biomass allocation (through phloem flux). The tree biomass is distributed among the plant organs: the leaves, the branches, the roots and the fruits. All along its life, a tree never stops creating and losing new organs so that its biomass is constantly changing over time and space. These dynamics are not well known at the scale of the plant nor the plot, but the above-ground biomass of a tree can be non-destructively monitored. The purpose here is to see how the CEDI workflow could help to characterize the dynamics of the above-ground tree biomass. So, from this angle of view, we define the variable of the PDE as the above-ground biomass of a tree and we note it b, like biomass.

Mathematically speaking, the variable b varies over a space domain Ω in $R^{3}$ encompassing the tree and over a time frame ]0,T] with T>0, where T is the lifespan of the tree. That is, we consider b as a function b:Ω×[0,T]→R. We assume that b satisfies sufficient regularity assumptions. We denote $x=\left(x_{1},x_{2},x_{3}\right)\in R^{3}$ the space coordinates in Ω associated with the Cartesian frame $\left\{\vec{e_{1}},\vec{e_{2}},\vec{e_{3}}\right\}$ in $R^{3}$ and t the time coordinate between 0 and T. Assuming that b is differentiable with respect to $x_{1}$, $x_{2}$ and $x_{3}$ (up to order k) and denoting $\gamma =\left(\gamma_{1},\gamma_{2},\gamma_{3}\right)\;\in \;{N^{+}}^{3}$ a multi-index of order $k=\left|\gamma \right|=\gamma_{1}+\gamma_{2}+\gamma_{3}\geq 1$, we introduce the notation $D^{\gamma }b\left(x\right)≔\frac{\partial^{\left|\gamma \right|}b\left(x\right)}{\partial_{x_{1}}^{\gamma_{1}}\partial_{x_{2}}^{\gamma_{2}}\partial_{x_{3}}^{\gamma_{3}}}$ of a $k^{th}$-order partial derivative of b and $D^{k}b\left(x\right)≔\left\{D^{\gamma }b\left(x\right)\;such\;that\;\left|\gamma \right|=k\right\}$ the set of all the $k^{th}$-order partial derivatives of b. We assume that the dynamics of b can be described by a partial differential equation of the form:

(1) $$\begin{array}{c} \frac{\partial b\left(x,t\right)}{\partial t}=F_{\beta }\left(D^{k}b\left(x,t\right),D^{k-1}b\left(x,t\right),\cdots ,\mathrm{Db}\left(x,t\right),b\left(x,t\right),x\right),\;\forall x\in \Omega \;\text{and}\;0<t\leq T, \end{array}$$

where the right hand side is an application $F_{\beta }:{R^{3}}^{k}\times {R^{3}}^{k-1}\times \ldots \times R^{3}\times R\times \Omega \to R$ that depends on n real parameters stored as a vector $\beta \in R^{n}$ with $n\in N^{+}\text{and}\;n\geq 1$.

Denoting by ∂Ω the boundary of Ω, the function b also satisfies boundary and initial conditions respectively given by b(x,t)=0,∀(x,t)∈∂Ω×]0,T] and $b\left(x,0\right)=b_{0}\left(x\right),\forall \left(x,t\right)\in \Omega \times \left\{0\right\}$ where $b_{0}$ is a known function defined from Ω to R.

At this stage of the workflow, we do not know the true form of the right-hand side of Eq. (1). If the list of partial derivatives is a priori known, the problem reduces to calibrate the PDE model, that is to find the value of β. According to [5] and [4], possible candidate terms for $F_{\beta }$ could be the biomass and its first and second order partial derivatives which gives the classical advection-reaction-diffusion operator:

$$\frac{\partial b}{\partial t}=\rho b-v\cdot Db+D\cdot \left(dDb\right)\;in\;\Omega \times ]0,T]$$

With $D≔{\left(\frac{\partial }{\partial x_{1}},\;\frac{\partial }{\partial x_{2}},\;\frac{\partial }{\partial x_{3}}\;\right)}^{T}$ the gradient vector, ρ∈R the parameter of reaction (or growth rate), $v={\left(v_{1},\;v_{2},\;v_{3}\;\right)}^{T}\in R^{3}$ the advection (or transport) vector which is homogeneous with a velocity and $d=diag\left(d_{1},d_{2},d_{3}\right)\in M^{3\times 3}\left(R\right)$ the matrix of diffusion where $d_{i}\in \;R^{+}\;$for i=1,2,3. In that case, there are seven real valued parameters $\beta ={\left(\rho ,v_{1},v_{2,}v_{3},d_{1},d_{2},d_{3}\right)}^{T}$ to determine. In the general context of tree biomass, we do not have access to these parameters’ values through direct measurements, but we do have estimates of the biomass b that can be used to deduce the values of the parameters β.

**Fig. 2:**
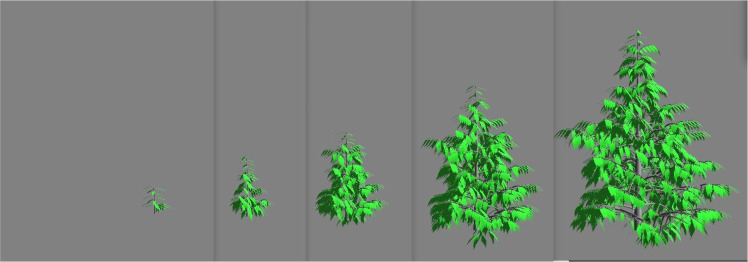
Tree growth of Massart type. Mock-ups obtained by the AmapSim software.

**Fig. 3:**
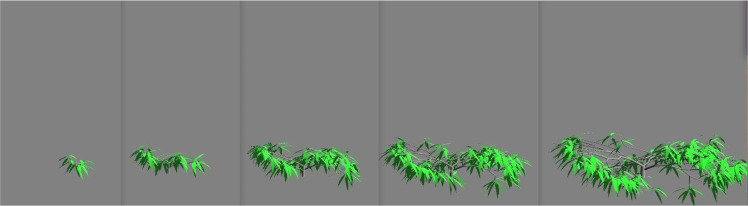
Tree growth of Prevost type. Mock-ups obtained with the AmapSim software.

**Fig. 4:**
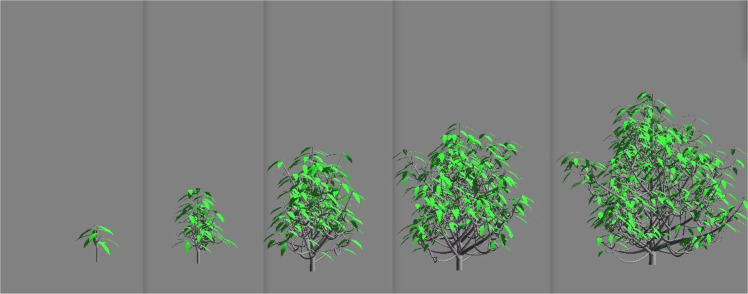
Tree growth of Rauh type. Mock-ups simulated by the AmapSim software.

Still for the sake of illustration and as a proof of concept of the workflow, we choose to build a biomass dataset from 3D digital mock-ups simulated by the dedicated software AmapSim [2]. This software aims at representing plant growth according to botanical knowledge on the fine architecture of the trees [8] and its variability (in the sense of [12]). The software generates 3D plant shapes (Fig. 2, Fig. 3, Fig. 4) accurate to reality in terms of organ numbers and organization over time. Based on those mock-ups, we virtually performed a set of $p\in N^{+}$ measurements of the biomass. Each measurement consists in a triplet $\left(b_{i}^{m},x_{i}^{m},t_{i}^{m}\right)$ where $b_{i}^{m}$ is the $i^{th}$ biomass value at the $x_{i}^{m}$- coordinates at time $t_{i}^{m}$. The resulting dataset is available at [1].

#### Extrapolation

In view of the computation of the parameters β, we need a tool, let say a surrogate model, to compute extrapolated values of the biomass at any space-time points. Let $b_{\alpha }^{s}$ denotes that surrogate model which depends on parameters α. The surrogate model $b_{\alpha }^{s}$ is built such that its parameters $\alpha =\left(\alpha_{1},\ldots ,\alpha_{N}\right)\in R^{N}$ minimize the following loss function

$$J_{1}\left(\alpha \right)=\frac{1}{2}\sum_{i=1}^{p}{\left|b_{\alpha }^{s}\left(x_{i}^{m},t_{i}^{m}\right)-b_{i}^{m}\right|}^{2}+\frac{c_{\alpha }}{2}\|\alpha {\|}_{2}^{2}$$

with $c_{\alpha }\geq 0$ a coefficient of regularization. For example, $b_{\alpha }^{s}$ can be a polynomial interpolation like in [17] or a spline interpolation where α stands for the coefficients in the polynomial or spline basis. The alternative we choose in this work was to define the surrogate model $b_{\alpha }^{s}$ as a multilayer perceptron used as a universal approximator like in [3] and in [16]. In that case α stands for the neural network coefficients and they are computed by neural network training. After training, thanks to $b_{\alpha }^{s}$, we can estimate the value of b at any points x∈Ω and at any time t∈]0,T]: $b_{\alpha }^{s}\left(x,t\right)\approx b\left(x,t\right)$.

#### Differentiation

The differentiation step gives tools to estimate values of the partial derivatives of b at any point in order to build a catalogue of possible candidate terms for $F_{\beta }$. Obviously, the computational method for the differentiation is related to the nature of the surrogate model. For example, in [17] the extrapolation and hence the partial derivative estimates are performed through a polynomial interpolation technique. In our case, the surrogate model is based on neural networks and the partial derivatives are obtained by back-propagation or automatic differentiation like in [3] and in [16]. Let us denote by $\hat{F_{\beta }}\;\left(b_{\alpha }^{s}\left(x,t\right)\right)$ an approximation of $F_{\beta }\left(b(x,t)\right)$ the right-hand side of Eq. (1) computed at point (x,t) thanks to the surrogate model $b_{\alpha }^{s}$.

#### Identification

**Fig. 5:**
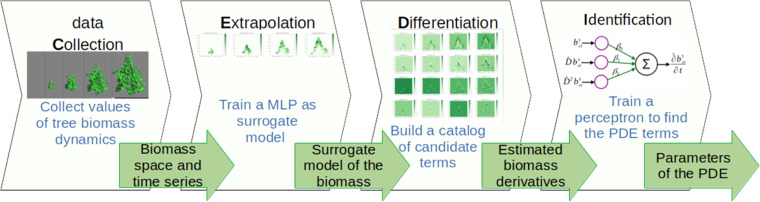
Data flowchart summarizing the CEDI workflow applied to tree biomass dynamics.

All the ingredients are almost there to perform the identification step which consists in solving the following optimization problem. Let us consider ${\left(x_{j},t_{j}\right)}_{j=1,q},$ a set of q>0 points in $\bar{\Omega }\times \left[0,T\right]$, not necessarily the same points as the p data points. Eq. (1) is satisfied at any of those points, and we can find the parameters β that best fit the data by minimizing the following loss function:

$$J_{2}\left(\beta \right)=\frac{1}{2}\sum_{j=1}^{q}{\left|\frac{\hat{\partial }b_{\alpha }^{s}\left(x_{j},t_{j}\right)}{\partial t}-\hat{F_{\beta }}\;\left(b_{\alpha }^{s}\left(x_{j},t_{j}\right)\right)\right|}^{2}+c_{\beta }{\left|\beta \right|}_{1}^{2}$$

where $\frac{\hat{\partial }b_{\alpha }^{s}\left(x_{j},t_{j}\right)}{\partial t}$ is an approximation of the time derivative of b at $\left(x_{j},t_{j}\right)$ computed from the surrogate model $b_{\alpha }^{s}$ and $c_{\beta }>0$ is a real parameter. The second term with 1-norm helps to preserve the sparsity of β when there are many candidate terms in the searched PDE. For our work, we chose to implement this optimization problem thanks to a perceptron having the candidate terms as entries and the time partial derivative as output. Forcing the bias to zero, the values of the perceptron coefficients after training corresponds to the values of the parameters β, ensuring that the resulting PDE is human readable. Fig. 5 summarizes the four steps of the workflow applied to tree biomass, focusing on the data that travel from one step to the next.

Many expert choices must be made to perform the calculation described above: the neural network architecture and hyperparameters, the design of the loss function, the algorithm to minimize the loss function, the method to compute the partial derivatives for examples. These choices are not easy to make: results obtained with different methods might not be the same. And it still raises theoretical questions and implementation issues [9].

## Method validation

There is no direct way to validate the outputs of the workflow since the governing equations for plant biomass dynamics are not explicitly known. Instead, we monitored the results’ validity at each step of the workflow through convergence analysis and consistency with biomass dynamics. In the following, our purpose is 1) to illustrate that the outputs of the workflow are in accordance with the qualitative behavior of the biomass dynamics and 2) to show the workflow can provide quantitative metrics to characterize the dynamics of plants’ biomass.

### Experimental settings

We implemented the CEDI workflow in a Python3 script using Tensorflow 2.17 and Keras 3.5. We ran the workflow on CPUs of the MESO@LR-Platform at the University of Montpellier. We used datasets showing the dynamics of three trees of contrasted forms: Massart, Prevost and Rauh architectural types [12]. For each tree, we tested the workflow with four different catalogues or, in other words, four distinct sets of candidate terms for the PDE. We also run the computations with different batch sizes for training. We monitored the loss function during training, we visualized the predicted values of the biomass and compared the obtained PDE models.

#### The data

The datasets taken from [1] gather tree biomass values on cubic meshes with step sizes $\delta x_{1}=\delta x_{2}=\delta x_{3}=20$ cm from zero to 10 years after seedling with time step δt=0.1 year. We built these voxelized data from 3D mockups simulated with the AmapSim software [2]. Examples of these mockups are shown on Fig. 2 for the Massart tree, Fig. 3 for the Prevost tree and Fig. 4 for the Rauh tree at t=2, 4, 6, 8 and 10 years after seedling. To ensure the convergence of the training, the dataset has been normalized and scaled according to the following transformations. The normalized biomass has been computed such that $B=\frac{b}{\sigma_{b}}$ where b is the biomass in gram (g) and $\sigma_{b}$ is the standard deviation of the biomass values. The time and space coordinates have been scaled as follows: $\tau =\frac{t-\bar{t}}{\sigma_{t}}$ and $\xi_{i}=\frac{x_{i}-{\bar{x}}_{i}}{\sigma_{i}},i=1,2,3$ where $\bar{t}$ and ${\bar{x}}_{i}$ are the mean values of time and space coordinates and $\sigma_{t}$ and $\sigma_{i}$ are their standard deviations.

#### Details of the implementation

The surrogate model of the extrapolation step is a feed-forward neural network made of sequential layers starting with an input layer of four entries for $x_{1}$, $x_{2}$, $x_{3}$ and t followed by a 16-node dense layer and then three 32-node dense layers and finally the last layer with one output standing for the biomass. All the internal layers have hyperbolic tangent activation functions and L2-kernel regularization with coefficient set to $10^{-6}$. Preliminary tests showed that higher values (between $10^{-5}$ and $10^{-3}$) tend to erase the spatial and temporal variability of the predicted biomass field, and values higher than $10^{-2}$ fail to converge when training the surrogate model. The choice of hyperbolic tangent is motivated by regularity assumptions required for the differentiation step. The training was performed with Adam optimizer with learning rate set to $10^{-4}$ and Keras default values for other parameters. A learning rate higher than $10^{-3}$ gave unstable training in preliminary tests, while lower than $10^{-5}$ led to slow convergence. We trained the network with batch size equal to 2, 16 and 64 and observed the results after 100 epochs. In the derivation step, we computed estimates of first and second order derivatives of B with the Tensorflow function GradientTape using automatic differentiation technique. We computed the derivatives of B on the same space and time points as those of the dataset. And for the identification step, we build 4 different catalogues, numbered from Ⅰ to Ⅳ, of candidate terms given in Table 2. These terms are chosen in accordance with available expertise on plant biomass dynamics, see for example [5] and [4]. We assume that a tree may be measured through its global biomass B that varies along time and space due to tree growth in height and diameter (growth speed may change over time and direction). These variations depend on the tree species. Translated to mathematical terms, we constructed 4 operator catalogs based on the biomass B and its partial derivatives expressed either with Cartesian coordinates or cylindrical coordinates. The latter may offer a good fit since the trees represented in the dataset are roughly axially symmetrical around ${\vec{e}}_{3}$ axis. The term B is associated with exponential growth, the first order derivatives are associated with transport phenomena and the sum of the second order derivatives is associated with isotropic growth speed of the tree crown. Each catalogue lists a different combination of possible candidate terms for the PDE to be discovered. Catalog I fits with exponential biomass growth, vertical growth velocity and isotropic growth speed of the crown. Catalog II fits with exponential biomass growth, vertical/lateral growth velocity and isotropic growth speed of the crown. Catalog III fits with exponential biomass growth, vertical and radial growth velocity and crown isotropic growth speed in cylindrical coordinates. Catalog IV is equivalent to catalog II with cylindrical coordinates.

**Table 2:** List of the candidate terms for the 4 catalogues tested.

	Candidate terms for the PDE	Labels of the associated weights in Table 3
Catalogue Ⅰ	B;$\frac{\partial B}{\partial x_{3}}$; $\frac{\partial^{2}B}{\partial x_{1}^{2}}+\frac{\partial^{2}B}{\partial x_{2}^{2}}+\frac{\partial^{2}B}{\partial x_{3}^{2}}$	$\rho ;v_{3};d$
Catalogue Ⅱ	B;$\frac{\partial B}{\partial x_{1}}$; $\frac{\partial B}{\partial x_{2}};\frac{\partial B}{\partial x_{3}}$; $\frac{\partial^{2}B}{\partial x_{1}^{2}}+\frac{\partial^{2}B}{\partial x_{2}^{2}}+\frac{\partial^{2}B}{\partial x_{3}^{2}}$	$\rho ;v_{1};v_{2};v_{3};d$
Catalogue Ⅲ	B;$\frac{\partial B}{\partial r};$$\frac{\partial B}{\partial x_{3}}$; $\frac{\partial^{2}B}{\partial x_{1}^{2}}+\frac{\partial^{2}B}{\partial x_{2}^{2}}+\frac{\partial^{2}B}{\partial x_{3}^{2}}$	$\rho ;v_{r};v_{3};d$
Catalogue Ⅳ	B;$\frac{\partial B}{\partial r}$; $\frac{1}{r}\frac{\partial B}{\partial \theta };$$\frac{\partial B}{\partial x_{3}}$; $\frac{\partial^{2}B}{\partial x_{1}^{2}}+\frac{\partial^{2}B}{\partial x_{2}^{2}}+\frac{\partial^{2}B}{\partial x_{3}^{2}}$	$\rho ;v_{r};v_{\theta };v_{3};d$

Each catalogue gives rise to a different perceptron architecture: the inputs of the tested perceptrons are the terms listed in the catalogue. The output is always the time partial derivative. Again, these data are normalized to ensure a better convergence of the perceptron training. Let us denote Y the vector containing the values of $\frac{\partial B}{\partial t}$ (that is the perceptron’s output) and $X_{i}$ the vector containing the i^th^ candidate (that is the i^th^ perceptron’s input). The normalization are performed according to the following transformation: $Y^{*}=\frac{Y}{\sigma_{Y}}$ and $X_{i}^{*}=\frac{X_{i}}{\sigma_{X_{i}}}$ where $\sigma_{Y}$ and $\sigma_{X_{i}}$ are the standard deviation of Y and $X_{i}$ respectively. The perceptron was trained with Adam optimizer with learning rate set to $10^{-6}$ and batch size set to 2, 16 and 64. The value of the learning rate could have been increased, since values lower than or equal to $10^{-6}$ lead to slow convergence. A L1-regularization term was set to $10^{-2}$ for sparsity of the weights and the bias was forced to zero. Setting the L1-regularization term bellow $10^{-3}$ could have been a better choice, since this hyperparameter tends to nullify the values of the searched weights for values above $10^{-2}$.

### Experimental results

#### Convergence analysis and stability

**Fig. 6:**
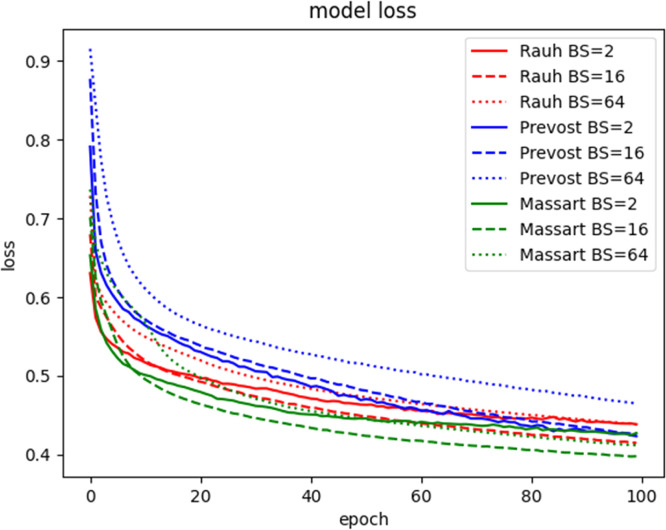
Convergence history of the extrapolation step for the data of Massart, Rauh and Prevost trees and batch size (BS) 2, 16 and 64.

Fig. 6 shows the convergence histories obtained when training the surrogate model (step Extrapolation). Convergence is almost reached after 100 epochs. For the Massart tree the smallest loss function value is obtained with a batch size of 16, while the biggest values are obtained with the Prevost tree for a batch size of 64. For the Rauh tree the 3 different values of the batch size seem to converge toward the same values of loss function. For the identification step performed with the catalogues of Table 2, we have seen on Fig. 11 that the batch size has a little impact on the value of the loss function. The batch size seems to impact only the speed of convergence. The learning rate which was set to $10^{-6}$ gave slow convergence: values between $10^{-5}$ and $10^{-3}$ could have led to faster convergence. Catalogues Ⅰ and Ⅱ, which are built with derivatives written in the Cartesian coordinate system, gave rather high values of the loss function, reaching plateau at values between 0.5 and 1.5. With the cylindrical coordinate system (catalogues Ⅲ and Ⅳ), the plateaus are slightly lower than in the Cartesian case. However, the batch size impacts the values of the coefficients (or weights) computed during the identification step as shown on Table 3. For example with catalogue number Ⅱ, from one value of the batch size to the other, the values of $v_{1}$ and $v_{2}$ can be totally different. This can be due to several reasons related to the data themselves or the algorithm. The space and time steps of the data might not be fine enough to generate a better solution space and the batch size and batch contents obtained after training data shuffling might not capture some data homogeneity. The data structure, or the loss function, or the starting guess of the optimizer, or the conjunction of all these reasons result in a solution space with many local minima. The same observation can be done for $v_{\theta }$ in catalogue Ⅳ, while catalogue Ⅲ leads to the convergence of $v_{r}$ and seems to be a better choice in terms of solution space. A comprehensive study examining the influence of batch size and optimization settings on PDE identification was deferred to future work, though such analysis would greatly enhance the reproducibility and robustness of our study.

**Table 3:** Results of the Identification step for catalogues (Cat) Ⅰ to Ⅳ.

	Batch size 2			Batch size 16			Batch size 64		
Cat I	Massart	Prevost	Rauh	Massart	Prévost	Rauh	Massart	Prévost	Rauh
ρ	0.171513	0.125952	0.108300	0.144744	0.356281	0.123811	0.182230	0.557582	0.180562
$v_{3}$	1.636139	7.886762	12.502697	2.081908	14.352541	16.067635	1.057330	23.830688	15.047523
d	0.000007	0.000001	0.000002	0.000036	0.012792	0.000114	0.003363	0.038559	0.005950
Cat II	Massart	Prevost	Rauh	Massart	Prevost	Rauh	Massart	Prevost	Rauh
ρ	0.172407	0.124174	0.105447	0.144677	0.435770	0.123829	0.183556	0.659764	0.199495
$v_{1}$	-0.007371	-0.030532	0.015476	0.003161	0.097990	-0.026351	-0.009714	0.240100	0.036013
$v_{2}$	1.159408	-3.257215	-2.676747	0.816525	6.716690	0.142282	1.438752	13.936645	3.062459
$v_{3}$	1.665916	7.781262	11.964144	2.046030	19.217773	15.933753	1.228995	27.171227	16.209906
d	0.000005	0.000010	0.000005	0.000038	0.015663	0.000099	0.003496	0.043053	0.007138
Cat III	Massart	Prevost	Rauh	Massart	Prévost	Rauh	Massart	Prévost	Rauh
ρ	0.023123	0.043417	-0.000052	0.000280	0.132076	0.001193	0.013196	0.404871	0.038897
$v_{r}$	0.255366	0.295080	0.161790	0.255238	0.316948	0.162605	0.276321	0.325743	0.194013
$v_{3}$	-2.018375	7.817176	10.520422	-0.921041	9.668255	13.520478	-2.552996	17.761522	12.505530
d	-0.000000	-0.000000	0.000000	-0.000073	0.002752	-0.000071	0.000342	0.017509	0.003785
Cat IV	Massart	Prevost	Rauh	Massart	Prévost	Rauh	Massart	Prévost	Rauh
ρ	0.021942	0.042844	-0.002183	0.000322	0.144933	0.001171	0.014234	0.440772	0.062709
$v_{r}$	0.256145	0.292236	0.162157	0.255994	0.312831	0.168146	0.275941	0.305816	0.180212
$v_{\theta }$	-0.470653	0.532653	1.010458	-0.209571	2.873393	-0.866930	-0.135464	6.037452	0.577699
$v_{3}$	-2.080043	7.960285	10.107252	-0.971721	10.907381	13.683924	-2.532960	21.319525	12.272537
d	-0.000003	0.000002	-0.000000	-0.000075	0.003444	-0.000065	0.000390	0.018244	0.004817

#### Validity of the results with respect to biomass dynamics

**Fig. 7:**
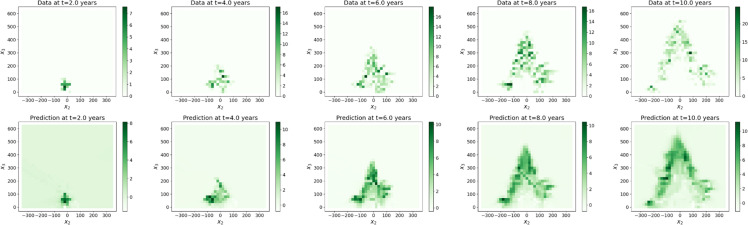
Biomass of the Massart tree plotted on the $\left(O,{\vec{e}}_{2},{\vec{e}}_{3}\right)$-plane: data (top) and predicted values (bottom) by the extrapolation with the surrogate model (trained with a batch size of 2).

**Fig. 8:**
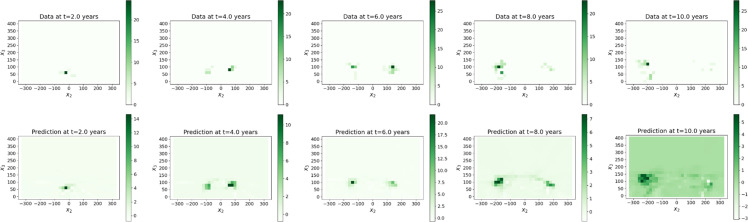
Biomass of the Prevost tree plotted on the $\left(O,{\vec{e}}_{2},{\vec{e}}_{3}\right)$-plane: data (top) and predicted values (bottom) by the extrapolation with the surrogate model (trained with a batch size of 2).

**Fig. 9:**
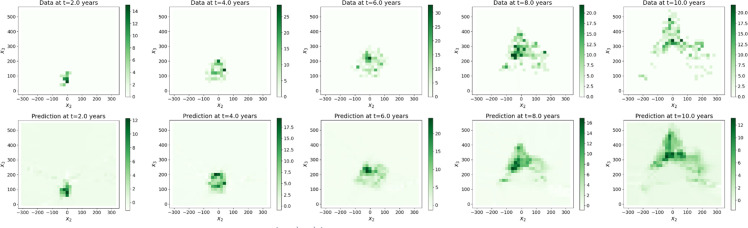
Biomass of the Rauh tree plotted on the $\left(O,{\vec{e}}_{2},{\vec{e}}_{3}\right)$-plane: data (top) and predicted values (bottom) by the extrapolation with the surrogate model (trained with a batch size of 2).

**Fig. 10:**
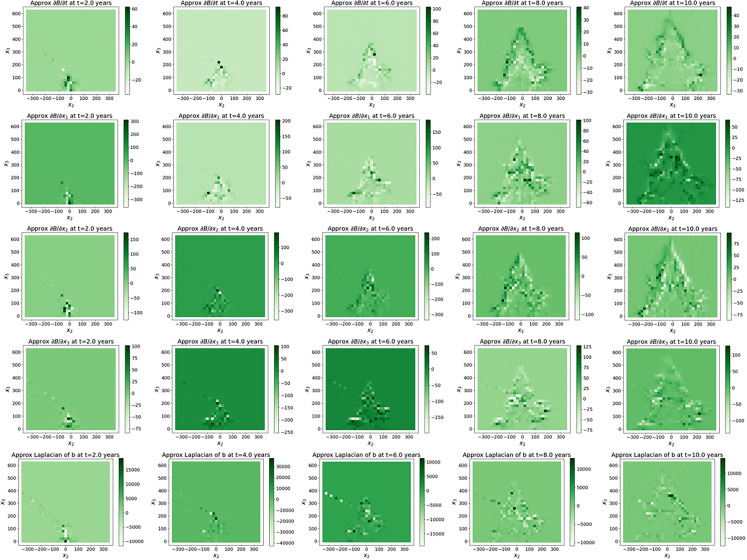
Massart tree. Plots on the $\left(O,{\vec{e}}_{2},{\vec{e}}_{3}\right)$-plane of isovalues of the biomass partial derivatives associated with catalogue Ⅱ. The first line shows isovalues of $\frac{\partial B}{\partial t}$ at t=2, 4, 6, 8 and 10 years (from left to right). The following lines show isovalues of $\frac{\partial B}{\partial x_{1}}$, $\frac{\partial B}{\partial x_{2}}$, $\frac{\partial B}{\partial x_{3}}$ and $\frac{\partial^{2}B}{\partial x_{1}^{2}}+\frac{\partial^{2}B}{\partial x_{2}^{2}}+\frac{\partial^{2}B}{\partial x_{3}^{2}}$ respectively at t=2, 4, 6, 8 and 10 years (from left to right).

The values of the tree biomass predicted by the surrogate models (step Extrapolation) inside the plane $\left(O,{\vec{e}}_{2},{\vec{e}}_{3}\right)$ shown on Fig. 7, Fig. 8, Fig. 9 are visually in accordance with the data. Besides, the mean absolute errors between the predicted scaled biomass and the scaled data were equal to 0.13 for Rauh and Massart, while they were equal to 0.26 for Prevost with the three different batch sizes. Some of the partial derivatives computed by automatic differentiation are shown on Fig. 10 for the Massart tree. Again, the plots show the values inside the $\left(O,{\vec{e}}_{2},{\vec{e}}_{3}\right)$-plane. Visually, the first order derivatives are consistent with the biomass dynamics. The second order partial derivatives seem to be blurred by numerical errors which may affect the rest of the workflow. The computed parameters can be interpreted with respect to the biomass dynamics. For all the trees and all the catalogues tested, the growth rates ρ associated with exponential growth of the biomass is rather small, ranging between almost zero and 0.6 per year. Exponential growth might not be a good choice for these datasets, instead we could have considered logistic growth for example. Similarly, the diffusion d which is associated with the second order derivatives is really small. This might be due to the fact that either diffusion occurs with really small amplitude or the second order derivatives are poorly approximated (see Fig. 10). Additional runs should be performed with different hyperparameter values, in particular with a smaller L1-regularization term, to verify whether the diffusion still cancels. The transport of the biomass along the vertical axis ($v_{3}$) also called vertical velocity is well captured for the Prevost and Rauh trees. The values of $v_{3}$ are higher for Rauh than for Prevost, which is in accordance with the fact that the Rauh tree reached higher altitude than Prevost after 10 years of growth: 5 meters against less than 2 meters. With these values, we would have expect a vertical velocity ($v_{3}$) of around 50 centimeters per year for Rauh and 20 centimeters per year for Prevost in the case of continuous growth in time, while the computations gave smaller values of $v_{3}$ for all the trees. These differences might be due to the fact that the transport term captures variations in leaf biomass and leaf turnover along the life of the trees. Catalogues Ⅰ and Ⅲ gave better results (in particular for the transport parameters) than catalogues Ⅱ and Ⅳ respectively in terms of stability. This can be interpreted at the biomass dynamics level by noticing that lateral transport of the biomass occurs roughly, first along the vertical axis and second, along all radial directions with approximately the same amplitude.

**Fig. 11:**
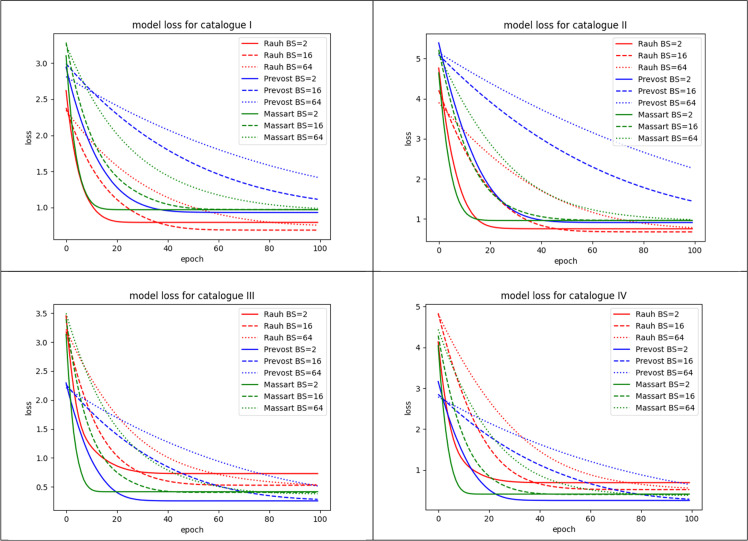
Convergence histories of the identification step with catalogues Ⅰ to Ⅳ for Massart, Prevost and Rauh trees with batch sizes (BS) 2, 16 and 64.

## Limitations

Each of the four steps of the CEDI workflow raises modeling questions as well as theoretical and technical issues that we did not address in the paper but that must be considered for future works. Since every step depends on the previous one, the final result may be influenced by some error propagation and modeling choices. Given the CEDI workflow, these issues may probably strongly depend on the PDE that has to be discovered and the kind of subject under study. The dataset processed during the first step (C) must be a relevant representation of the dynamics of interest. A trade-off between representativity, cost of acquisition/curation and accuracy of the data must be handled. In our work, even though the data space-time sampling gives a good representation of the dynamics of the biomass (since they are generated thanks to a dedicated software) we encountered some stability issues that could be worse in cases of data collected in the field. Acquiring data with such small space and time sampling steps can be a tedious and costly task, unless one uses remote sensing tools for example. The challenge with the design of the surrogate model during the second step (E) is then to compute missing data that is in line with the dynamics under study. In addition, the setting of the surrogate model raises issues about the choice and the implementation of an extrapolation method, which is expected to give a faithful representation of the dynamics at any space-time point. Computing estimates of partial derivatives (step D) requires that the surrogate model is differentiable, which has not been proved here (and which is not necessarily always the case). With the neural network approach, we rely on the automatic differentiation tools implemented in deep learning libraries, but we did not estimate the numerical errors made. The identification step (I) also raises theoretical and implementation issues. This kind of problem is often ill-posed and convergence and stability may be analyzed carefully. Implementation issues particularly deal with the management of boundary conditions, the design of a neural network architecture and the setting of a successful optimizer. As each step depends on the previous one, an accumulation of errors can occur, which can lead to a lack of stability or bad convergence rate in the identification step. Studies need to be conducted to evaluate the error propagation behavior inside the CEDI workflow. For each specific case, this may be achieved by the collaboration of domain experts who provide multiple empirical datasets with analytical point of view and numerical experts who perform successive trials and tests on various settings along the four CEDI steps.

Through a simple numerical implementation, we showed that the CEDI workflow is able to capture the qualitative dynamical behaviour of trees’ biomass. However, our attempt for the modeling of tree dynamics is far from being perfect in view of the architecture but also the physiology of the plants. The work presented in the paper has only the merit of illustrating the workflow in a concrete case, as proof of concept and to shed light on the many difficulties encountered and improvements to be done. The dynamics of plants are complex in many and various aspects. Plants are living and growing organisms that can be studied at different times and spatial scales. In addition to all the intraspecific and interspecific variability linked to the plants themselves, the environment has a strong influence on plant growth and dynamics through soil conditions, water availability, air and soil temperatures, human practices, pests and diseases for example. Plant growth and dynamics are driven by many physiological, mechanistic, hydraulic and agronomic processes. Few modeling approaches link all these processes in a single model. Each process is associated with its specific type of data which has been massively collected for a very long time, making modeling even more complex, and integrating all that knowledge is a complex task. The CEDI workflow offers a generic tool that might open new roads for the characterization of the plant biomass dynamics provided meaningful datasets are available. We also are convinced that this genericity has to be carefully controlled and set up through a collaboration between experts on the current topic and experts on numerical studies.

To go further, future work concerns the application of the CEDI workflow to real-world field data, since the design of the workflow was originally motivated by the characterization of biomass dynamics in cocoa-based agroforestry systems. Remote sensing technologies such as airborne or terrestrial Lidar provide tools to monitor ecosystems at different scales. Lidar data of real trees consists of 3D point clouds (see for example the dataset described in [15]) whose use in the CEDI workflow could raise many challenges, particularly in the data Collection step. First, a Lidar scan provides static information at a single date while the life of a tree may last several decades. The monitoring of tree biomass dynamics along its life requires many scans performed at well chosen dates according to the phenology of the tree, which would lead to long and expensive data collection campaigns. An alternative based on a strong knowledge of the phenology would consist in reconstructing chrono-sequences of Lidar data from scans of different trees at different stages of their life, provided they all have grown in similar conditions. Another alternative would consist in morpho-architectural studies of scans of a single tree to reconstruct its past. Second, even though Lidar scans provide tree descriptions with high space accuracy, they suffer from artefacts such as occlusions caused by hidden vegetation or blurring due to winds. To limit occlusions, several scans have to be done at different positions around the same single tree, increasing the operating time in the field and the memory size of the data. To limit the blurring effect, the tree branches and leaves must not move between two scanning positions, requiring good weather conditions without wind nor rain. Third, as mentioned above Lidar data consists of 3D point clouds characterized by the list of point coordinates that have been hit by the laser ray of the Lidar scanner device. The development of algorithms dedicated to the analysis of such data is a research question in itself where the challenge is to assess traits such as woody biomass and foliage distribution from point cloud coordinates. Fourth, to tackle realistic applications that have a real interest for plant scientists, additional traits on top of the tree biomass must be taken into account since issues in the management of agro-ecosystems deal with yield, light availability, pest and disease control, climate change mitigation among others. These issues require applying the CEDI workflow not only at the tree scale but at the plot scale, which involves manipulating bigger and more complex datasets and leading to challenges linked to scaling up, potentially noisy and incomplete data. Finally, many back-and-forth loops between plant and digital experts would be needed to build meaningful dataset in relation to the scientific question and capture the dynamics with sufficient representativeness to enable stable and accurate numerical processing of steps E, D and I of the workflow.

## Conclusion

The main contribution of our work is to introduce a generic workflow for the complex task of discovering PDE models from data. Our workflow, called CEDI, is divided into four main steps: the data Collection, Extrapolation, Derivation and identification steps. The four steps encompass the task of gathering a meaningful dataset, the setting up of a surrogate model in case of missing data, the computation of partial derivatives and finally the extraction of the PDE that best matches with the dataset. The workflow CEDI offers a conceptual framework that unifies methodologies for extracting PDE from data, and has the advantage of being generic and flexible. The workflow is particularly useful for applications where theoretical models are incomplete or unavailable. As a preliminary candid test, we applied the workflow to the modeling of tree biomass dynamics using realistic data and a neural network approach. While this basic application gave promising results, future work should focus on improving the neural network architectures and a comprehensive study of the impact of the hyperparameters on the convergence and accuracy. The numerical results presented in this paper have the merit of illustrating the conceptual framework of the CEDI workflow and is a methodological milestone toward applications to real world data, to be performed in conjunction with plant and digital experts, in the perspective to address questions related to the understanding of plants’ dynamics. The workflow paves the way for cognitive tools to generate knowledge through data-driven PDE modeling within the paradigm of theory-driven data science, while enabling researchers from diverse fields to pool their efforts and structure their work in favor of the understanding of dynamical phenomenon in life sciences.

## Ethics statements

Our work did not involve human subjects, animal experiments or data collected from social media platforms.

## CRediT author statement

**Emilie Peynaud:** Conceptualization, Methodology, Software, Validation, Formal analysis, Writing - Original Draft, Writing - Review & Editing, Visualization, Supervision, Project administration, Funding acquisition. **Paulin Melatagia:** Conceptualization, Formal analysis, Methodology, Validation, Writing - Original Draft, Writing - Review & Editing. **Serge Stinckwich:** Conceptualization, Methodology, Writing - Original Draft, Writing - Review & Editing. **Jean-François Barczi:** Data production, Writing - Review & Editing, Resources, Data Curation, Visualization.

## Declaration of competing interest

The authors declare that they have no known competing financial interests or personal relationships that could have appeared to influence the work reported in this paper.

## Data Availability

Links to data and source code are given in the specifications table.
